# The Complete Chloroplast Genome of Endangered Species *Stemona parviflora*: Insight into the Phylogenetic Relationship and Conservation Implications

**DOI:** 10.3390/genes13081361

**Published:** 2022-07-29

**Authors:** Ran Wei, Qiang Li

**Affiliations:** 1 College of Life Science and Technology, Xinjiang University, Urumqi 830046, China; 17855023682@163.com; 2 Laboratory of Adaptation and Evolution of Plateau Biota, Northwest Institute of Plateau Biology, Chinese Academy of Sciences, Xining 810008, China

**Keywords:** *Stemona*, *Stemona parviflora*, chloroplast genome, phylogenetic, niche data

## Abstract

*Stemona parviflora* is an endangered species, narrowly endemic to Hainan and Southwest Guangdong. The taxonomic classification of *S. parviflora* remains controversial. Moreover, studying endangered species is helpful for current management and conservation. In this study, the first complete chloroplast genome of *S. parviflora* was assembled and compared with other *Stemona* species. The chloroplast genome size of *S. parviflora* was 154,552 bp, consisting of 87 protein-coding genes, 38 tRNA genes, 8 rRNA genes, and one pseudogene. The ψ*ycf1* gene was lost in the cp genome of *S. sessilifolia*, but it was detected in four other species of *Stemona.* The inverted repeats (IR) regions have a relatively lower length variation compared with the large single copy (LSC) and small single copy (SSC) regions. Long repeat sequences and simple sequence repeat (SSR) were detected, and most SSR were distributed in the LSC region. Codon usage bias analyses revealed that the RSCU value of the genus *Stemona* has almost no difference. As with most angiosperm chloroplast genomes, protein-coding regions were more conservative than the inter-gene spacer. Seven genes (*atpI*, *ccsA*, *cemA*, *matK*, *ndhA*, *petA*, and *rpoC1*) were detected under positive selection in different *Stemona* species, which may result from adaptive evolution to different habitats. Phylogenetic analyses show the *Stemona* cluster in two main groups; *S. parviflora* were closest to *S. tuberosa*. A highly suitable region of *S. parviflora* was simulated by Maxent in this study; it is worth noting that the whole territory of Taiwan has changed to a low fitness area and below in the 2050 s, which may not be suitable for the introduction and cultivation of *S. parviflora.* In addition, limited by the dispersal capacity of *S. parviflora*, it is necessary to carry out artificial grafts to expand the survival areas of *S. parviflora*. Our results provide valuable information on characteristics of the chloroplast genome, phylogenetic relationships, and potential distribution range of the endangered species *S. parviflora*.

## 1. Introduction

*S. parviflora*, belonging to the genus *Stemona*, is narrowly endemic to Hainan and Southwest Guangdong. It is born in valleys, streams, and stone cracks at an altitude of 700 m [1]. *Stemona* mainly relies on ants to spread seeds. Due to the limited diffusion ability of ants, the vast majority of Stemonaceae species cannot cross the barrier of the ocean and become a narrow endemic group [2,3]. Amplified Fragment Length Polymorphism (AFLP) analyses demonstrated that *S. parviflora* has high genetic diversity and low genetic differences. In addition to the insect vector, the water vector and wind vector may also appear in *S. parviflora* at the same time [4]. *S. parviflora* can be used as a medicine; its effective component is an alkaloid, which has an antitussive effect. Phytochemical studies have shown that 17 alkaloids and 22 non-alkaloids have been extracted from parts of *S. parviflora* [5,6]. Given its great economic value and the fact the limited population of *S. psrviflora* are threatened by habitat fragmentation by human activity, the *S. parviflora* was considered an endangered species (EN) in the Threatened Species List of China’s Higher Plants [7].

Taxonomic classification has remained controversial in *S. parviflora* due to the use of the different molecular markers. *S. parviflora* were separated from *S. japonica*, *S. sessilifolia,* and *S. tuberosa* and were more closely related to *S. phyllantha*, *S. aphylla*, *Stemona* sp. based on *trnH-psbA* cpDNA markers [8]. The phylogenetic analyses were based on five cpDNA (*atpI-atpH*, *psbB-psbH*, *rpl4-rpl36*, *trnC-ycf6*, *trnL-trnF*), implying that *S. parviflora* were sister to *S. javanica* with an extremely low bootstrap value of 52% [9]. Recent studies based on three cpDNA markers (*matK*, *rbcL*, *psbK-psbI*) [3] suggest that *S. parviflora* was sister to *S. tuberosa*.

Climatic oscillation plays a crucial role in the pattern of a species distribution area on historical, current, and future scenarios. Comparative history and the current potential distribution range can help to us comprehend the historical dynamic processes of the population [10]. Understanding future invasion ranges is crucial for nature conservation and management. Maximum entropy (MaxEnt) has been widely applied to predict the potential distribution area of many taxa, especially in economic crops and endangered species [11]. For example, MaxEnt modeling has excellently predicted the potential distribution range of *Mentha pulegium*, which shows a northeastward to center-eastward shift of suitable regions, and the high suitable areas would decrease in 2050 s and 2070 s compared with the current distribution, thus providing useful information for current management [12]. In addition, MaxEnt was more suitable for predicting the distribution area of a small population under current and future circumstances [13]. The distribution pattern of *S. parviflora* is relatively unknown. Identifying potential distribution areas of *S. parviflora* will be meaningful for current management.

The structure of the chloroplast genome is relatively conservative in land plants, with a large single copy (LSC), a small single copy (SSC), and two inverted repeats (IR) in most angiosperm plants [14]. However, chloroplast genome variation occurs in different plant taxa, even at an intraspecific level, e.g., the length of gene expanded to the boundary of IR/SSC and IR/LSC region vary in different degrees among genera, interspecies, and interspecific levels [15]. Gene loss and pseudogenization occur in many angiosperms. For example, the gene *infA* was lost from some genus of Aroideae and Lemnoideae [16]. The chloroplast genome is widely used in angiosperm phylogenetic analysis, species definition, and the inference of species’ divergence time and geographical origin due to its sequence conservation and parthenogenetic characteristics [17,18,19].

Studies on endangered species are significant for us to understand the population’s evolutionary history. In this study, the first complete chloroplast genome of the endangered species *S. parviflora* was assembled and compared with other *Stemona* species. Our main goals were: (1) to characterize and compare the chloroplast genome of genus *Stemona*; (2) to assess the taxonomic position of *S. parviflora*; and (3) to simulate the potential distribution range of *S. parviflora* and provide protective measures.

## 2. Materials and Methods

### 2.1. Sample Collection, DNA Sequencing, and Chloroplast Genome Assembly

The leaf materials of *S. parviflora* were collected from Bopian Village, Qiongshan District, Hainan Provence (19°55′ N, 110°20′ E). The silica gel-dried leaves of *S. parviflora* were sent to Genepioneer Biotechnologies (Nanjing, China) for sequencing. A tissue material of 30 mg was added to liquid nitrogen, and then DNA was extracted using the DP305 kit. The samples were sequenced on the NovaSeq 6000 sequencing platform, and the length of sequencing was 150 BP. High-quality clean data were obtained by removing low-quality sequences. The *S. parviflora* cp genome was assembled using the GetOrganelle v1.7.5.0 pipeline with the *Stemona japonica* cp genome as the reference [20]. The bandage was used to confirm that the assembly graph was a circle [21].

### 2.2. Chloroplast Genome Annoation

The annotation of *S. parviflora* cp genome was completed using the online program GeSeq https://chlorobox.mpimp-golm.mpg.de/geseq.html (accessed on 16 August 2021). Sequin software modifies the GenBank file of *S. parviflora*. The online tools at https://chlorobox.mpimp-golm.mpg.de/OGDraw.html (accessed on 16 August 2021) were used to visualize the structure map of the chloroplast genome. The cp genome of *S. parviflora* was submitted by the Banklt platform under the accession number MZ151339.

### 2.3. Condon Usage Bais and Repeat Sequence Analyses

The codon preferences of protein sequences encoded by five species of *Stemona* were counted by MEGA X software (https://www.megasoftware.net/dload_win_gui, accessed on 16 August 2021) [22]. The long repeat sequences were predicted by REPuter [23], with the minimum repeat size set at 30 and a Hamming distance of 3. MIcroSAtellite identification tool (MISA) software was used to check simple sequence repeats (SSRs), with 10, 5, 4, 3, 3, and 3 for mono-, di-, tri-, tetra-, and pentanucleotide sequences [24].

### 2.4. Comparative Analysis of the Chloroplast Genomes

The IR/SSC and IR/LSC junctions of *S. japonica*, *S. tuberosa*, *S. parviflora*, *S. mairei*, and *S. sessilifolia* were compared using the online tool Irscope (https://irscope.shinyapps.io/irapp/ (accessed on 12 June 2022)). The online tool mVISTA (https://genome.lbl.gov/vista/mvista/submit.shtml (accessed on 12 June 2022)) was used to compare the complete chloroplast genomes of the five species, with the annotation of *S. parviflora* as the reference.

### 2.5. Ka/Ks Value Analyses

To understand the natural selection pressure in the evolution of the genus *Stemona*, homologous protein sequences between *S. parviflora* and other *Stemona* species were obtained using BLASTN. Then, the shared protein-coding genes were aligned using MAFFT version 7 [25]. The non-synonymous (Ka) and synonymous (Ks) ratios (Ka/Ks) were calculated using KaKs_Calculator version 2 [26].

### 2.6. Phylogenetic Analysis

To explore the phylogenetic relationships of *S. japonica*, *S. tuberosa*, *S. parviflora*, *S. mairei*, and *S. sessilifolia*, 80 protein-coding genes were extracted, maffted, and concatenated by PhyloSuite [27]. Bayesian Inference (BI) analyses were conducted using MrBayes version 3.2.6 in PhyloSuite, which is under the GTR + F + G4 model (two parallel runs, 1,000,000 generations) from ModelFinder.

### 2.7. Ecological Niche Modeling

The longitude and latitude information of *S. parviflora* was collected from published research papers and the National Specimen Information Infrastructure (http://www.nsii.org.cn/2017/home.php (accessed on 23 June 2022)) (Table S1). After coordinate point redundancy analysis, 19 effective distribution information points were reserved for subsequent analysis. Nineteen environmental and climatic factors were downloaded from WorldClim https://worldclim.org/data/index.html (accessed on 23 June 2022), and ENMTools version 1.4.0 software was used for the correlation analysis of environmental factors [28]. A pairwise correlation comparison between current climate factors and future climate factors was carried out, respectively, and the climate factors with a correlation coefficient greater than 0.8 (*R*^2^ ≥ |0.8|) were removed. Eight current climate factors have been retained, including mean diurnal range/°C (Bio2), Isothermality (Bio3), temperature (standard deviation) seasonality (Bio4), minimum temperature of coldest month/°C (Bio6), precipitation of wettest month/mm (Bio13), precipitation of driest month/mm (Bio14), precipitation of warmest quarter/mm (Bio18), and precipitation of coldest quarter/mm (Bio19). Nine future climate factors were retained for predicting the future potential distribution range, including mean diurnal range/°C (Bio2), minimum temperature of coldest month/°C (Bio6), annual temperature range/°C (Bio7), mean temperature of wettest quarter/°C (Bio8), annual precipitation/mm (Bio12), precipitation of driest month/mm (Bio14), precipitation seasonality (Coefficient of Variation)/mm (Bio15), precipitation of warmest quarter/mm (Bio18), and precipitation of coldest quarter/mm (Bio19) (Table S2). Based on the selected climate factors, MaxEnt version 3.4.4 was used to simulate the current and future (2050 s) potential distribution areas of the *S. parviflora* [29]. Of the distribution point data, 80% was used for the training set and 20% was used for random detection. The operation was repeated 10 times, and the number of iterations was 5000.

## 3. Results

### 3.1. General Features of the Chloroplast Genome

**Figure 1:**
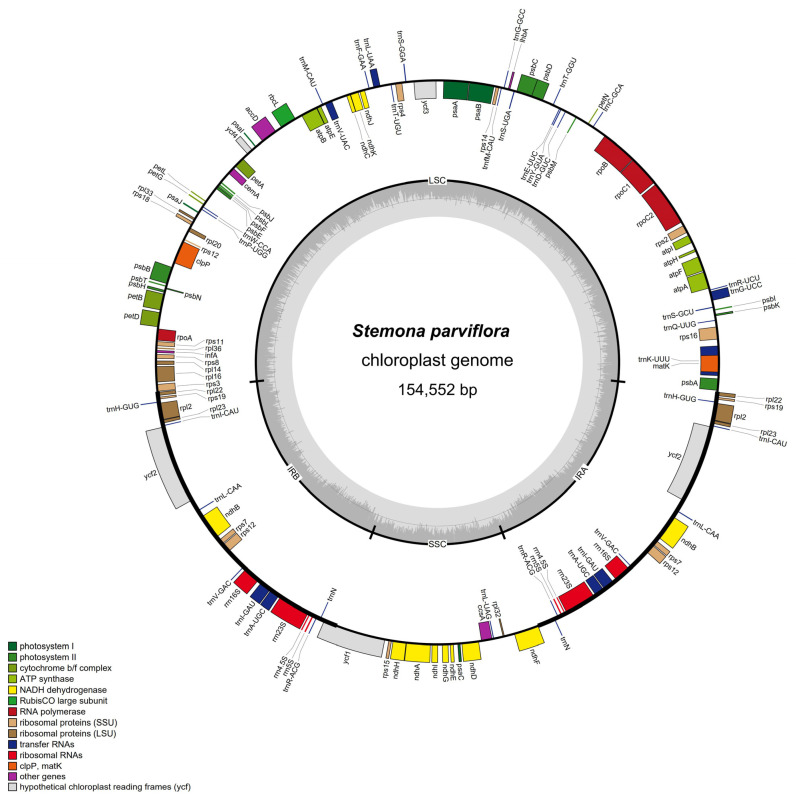
Chloroplast genome map of *Stemona parviflora*.

The chloroplast genome of *S. parviflora* had a conventional quadripartite structure characteristic of most land plants, with an LSC (82,410 bp), SSC (17,966 bp), and two IR regions (27,088 bp) (Figure 1). The length of the chloroplast genome of *Stemona* was about 15.4 KB, of which the longest size of the plastid genome was *S. parviflora* (154,552 bp). *S. sessilifolia* had the shortest chloroplast genome in size (154,037 bp). Among the five species of *Stemona*, the length difference of the LSC region was 461 bp, the length difference of the SSC region was 77 bp, and the length difference of IR regions was 22 bp. The IR regions had a relatively lower length variation compared with the LSC and SSC regions. The LSC region had a high variation in the length of the plastid genome. The GC content of *S. sessilifolia* and *S. japonica* was 38.0%; however, the GC content of *S. parviflora* and *S. tuberosa* accounted for 37.9%. *S. mairei* had the lowest GC content at 37.9%. The cp genome of *S. japonica*, *S. tuberosa*, *S. parviflora*, and *S. mairei* had 131 genes, including 87 protein-coding genes, 38 tRNA genes, 8 rRNA genes, and one pseudogene. One pseudogene was lost in the cp genome of *S. sessilifolia* (Table 1).

### 3.2. Long Repeat Sequence and SSR Analyses

**Figure 2:**
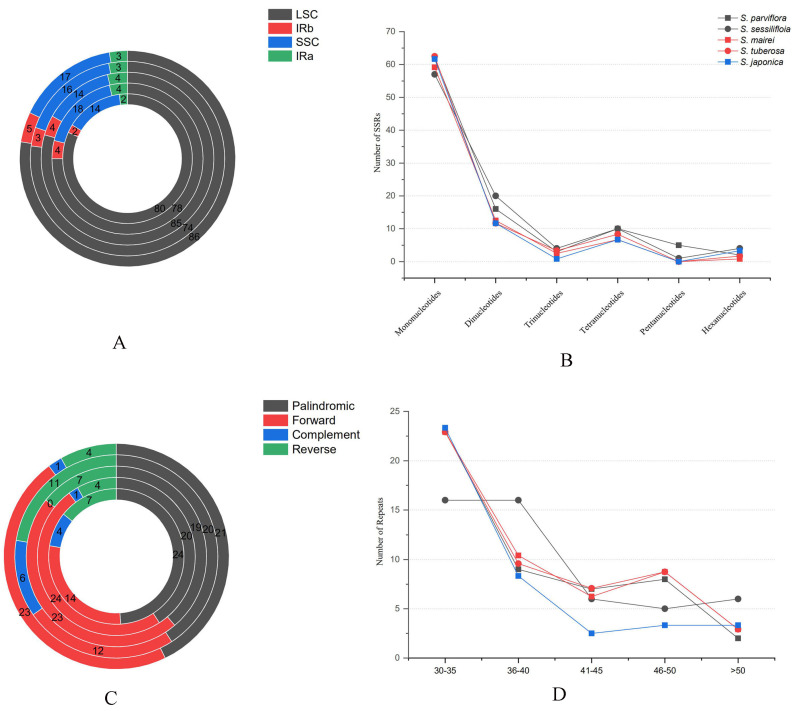
Distribution of repeats sequence in Stemona. (**A**) Number of SSR in the IR, SSC, and LSC regions; (**B**) Number of SSR in the five species of *Stemona*; (**C**) Number of four types of repeats in *Stemona*; (**D**) Length of four types of repeat sequences.

In total, 62, 16, 3, 4, 5, and 6 SSRs represented by mono-, di-, tri-, tetra-, penta-, and hexanucleotides repeats were found in *S. parviflora*, respectively. Of the SSRs, 98, 106, 107, 96, and 111 were detected in the *S. parviflora*, *S. sessilifloia*, *S. mairei*, *S. tuberose,* and *S. japonica* chloroplast genome (Figure 2A). The LSC region harbored the largest number of SSRs, followed by the SSC region. The IR regions (IRa and IRb) had a relatively lower number of SSRs compared with the LSC and SSC regions, as the majority of land plants’ mononucleotides’ repeats represent the most abundant of SSRs in the plastid genome (Figure 2B).

A total of 49 long repeats were identified in the cp genome of *S. parviflora*, including 24 palindromic, 14 forward, 4 complement, and 7 reverse repeats. The size of the long repeats in *S. parviflora* ranged from 30 to 56 bp (Figure 2C). The number of four types of long repeats was the same compared with other congeneric species. However, there was a significantly different number of palindromic, forward, complement, and reverse repeats, respectively. For example, no complement repeats were detected in the cp genome of *S. mairei*; the rest of the four species harbored four, one, six, and seven complement repeats, respectively. Moreover, most long repeats fell into 30–35 bp (Figure 2D).

### 3.3. Comparative Analysis of Chloroplast Genome of Stemona

**Figure 3:**
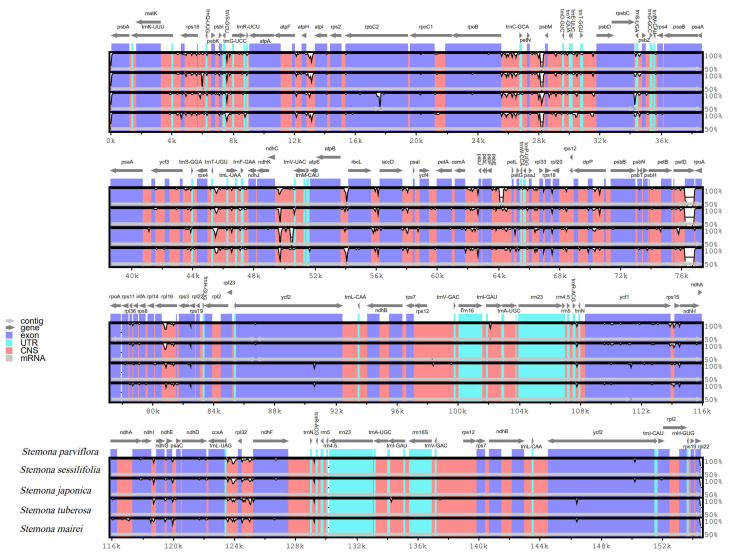
Comparison of *Stemona chloroplast* genomes using mVISTA, with *S. parviflora* chloroplast genome as reference.

The complete chloroplast genome sequence of *S. parviflora* was compared with congeneric species (*S. japonica, S. mairei, S. sessilifolia, and S. tuberosa*) using the online genome alignment tool mVISTA (Figure 3). Large sequence variations were detected between *S. parviflora* and the other congeneric species. These variations were mainly distributed in the LSC and SSC regions. In the protein-coding gene regions, chloroplast genome sequence variations mainly appeared in *rps16*, *trnQ-UUG*, *psbI*, *trnS-UGA*, *ycf3*, *trnS-GGA*, *trnL-UAA*, *psbJ*, *clpP*, *petD*, *rpl16*, *rpl22,* and *ycf1*. The most divergent regions in the intergenic spacer regions were found in *trnK-UUU_rps6*, *rps16_trnQ-UUG*, *trnS-GCU_trnG-UUC*, *atpF_atpH*, *atpH_atpI*, *rpoB_trnC-GCA*, *petN_psbM*, *psbM_trnD-GCU*, *trnT-UGU_trnL-UAA*, *ndhC_trnV-UAC*, *atpB_rbcL*, *rbcL_accD*, *accD_psaI*, *cemA_psbJ*, *psbE_petL*, *rpl20_rps12*, *ndhI_ndhJ*, *trnL-UAG_rpl32*, and *rpl32_ndhF*. Like most other land plans, the IR regions were more conservative than the LSC and SSC regions.

### 3.4. IR Expansion and Contraction

**Figure 4:**
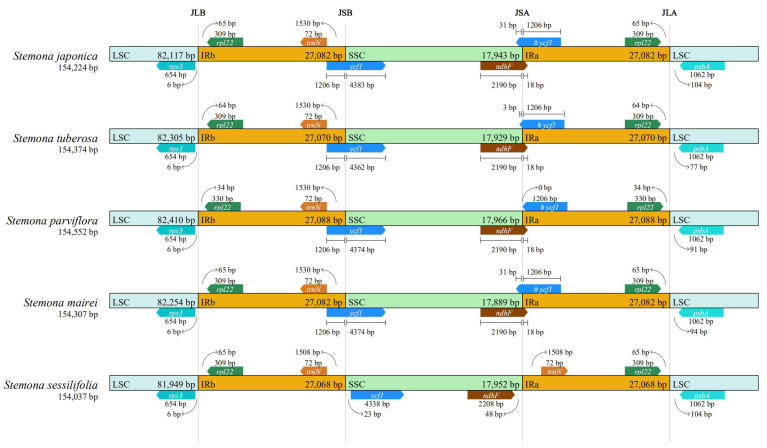
Comparisons of LSC, SSC, and IRs junctions among *Stemona* species.

The content and the number of genes in the boundary of IR/LSC and IR/SSC regions were compared among the five species of the genus *Stemona* (Figure 4). Except for *S. sessilifolia*, the rest of the four species contained the same content and the same number of genes in the IR/LSC and IR/SSC boundary, including *rps3*, *rpl22*, *trnN*, *ycf1*, *ndhF*, ψ*ycf,1,* and *psbA*. The gene *rps3* is completely located in the LSC region with 6 bp away from the IRb region. The *rpl22* and *trnN* genes are distributed in the LSC region. The gene *ycf1* in *S. sessilifolia* was located in the SSC region with 23 bp away from IRb region; however, *ycf1* in the rest of the four species all spanned the IRb/SSC boundary with 1206 bp extending to the IRb region. Furthermore, one pseudogene, ψ*ycf1,* was lost in the chloroplast genome of *S. sessiliflora*.

### 3.5. Codon Usage Bais of Stenoma Species

**Figure 5:**
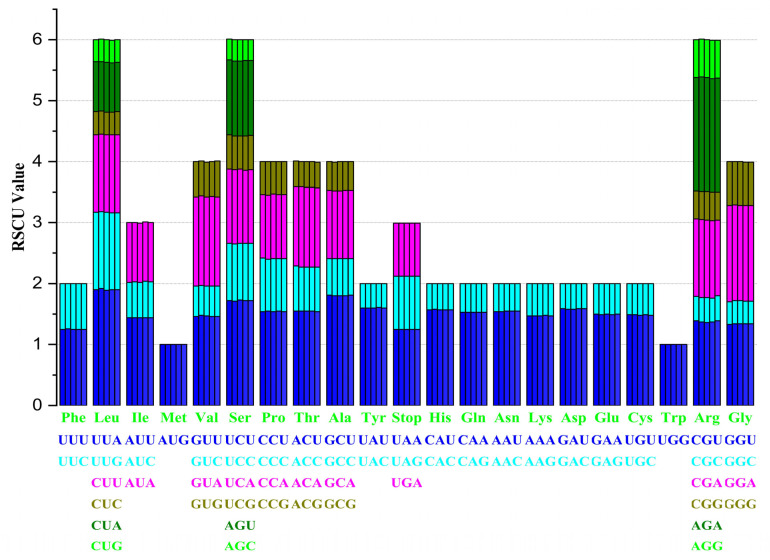
Codon distribution of protein-coding genes of the chloroplast genomes of *Stemona* species.

The average number of codons in *Stemona* was 22795. *Stemona japonica* had the largest number of codons at 22,808. The RSCU values of *S. japonica*, *S. parviflora*, *S. mairei*, and *S. sessilifolia,* all with 20 codons, were greater than 1, whereas the *S. tuberosa* with 29 codons was greater than 1. The A/U contents at the third codon position were the most preferred (Table S3). There was almost no difference in the RSCU value of *Stemona* species (Figure 5).

### 3.6. Selective Pressure Analyses

**Figure 6:**
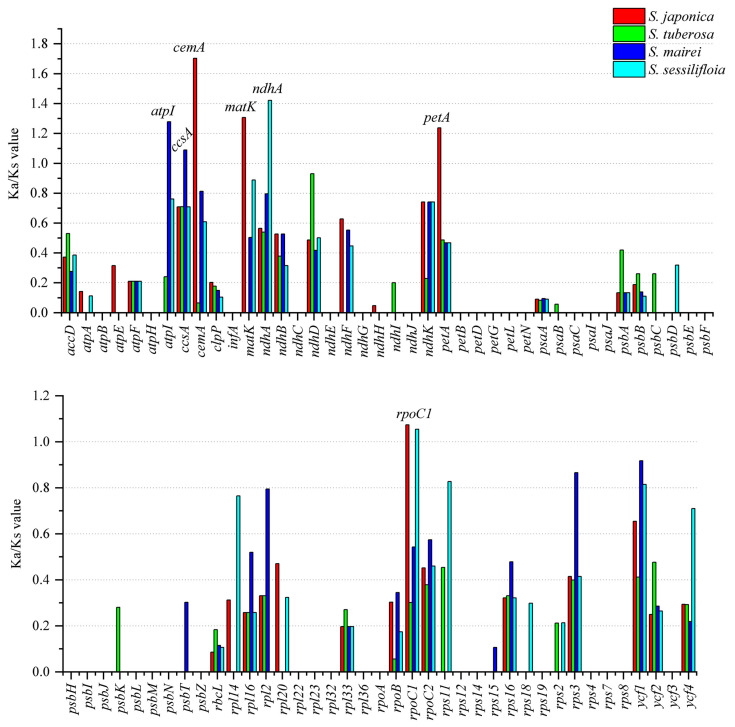
Detection of positive selection sites of chloroplast genes in *Stemona*, with *S. parviflora* as a reference genome.

The non-synonymous (Ka) and synonymous (Ks) ratio (Ka/Ks) was calculated among the protein-coding genes of *S. parviflora* and four *Stemona* species (*S. japonica*, *S. tuberosa*, *S. mairei*, and *S. sessilifloia*) (Figure 6). The majority of protein-coding genes with Ka/Ks were valued less than one, indicating that these genes are under the purifying selection effect. The Ks/Ks values of *atpI*, *ccsA*, *cemA*, *matK*, *ndhA*, *petA*, and *rpoC1* were greater than one, indicating that these genes are under positive selection.

### 3.7. Phylogenetic Relationships between S. parviflora and Other Stemona Species

**Figure 7:**
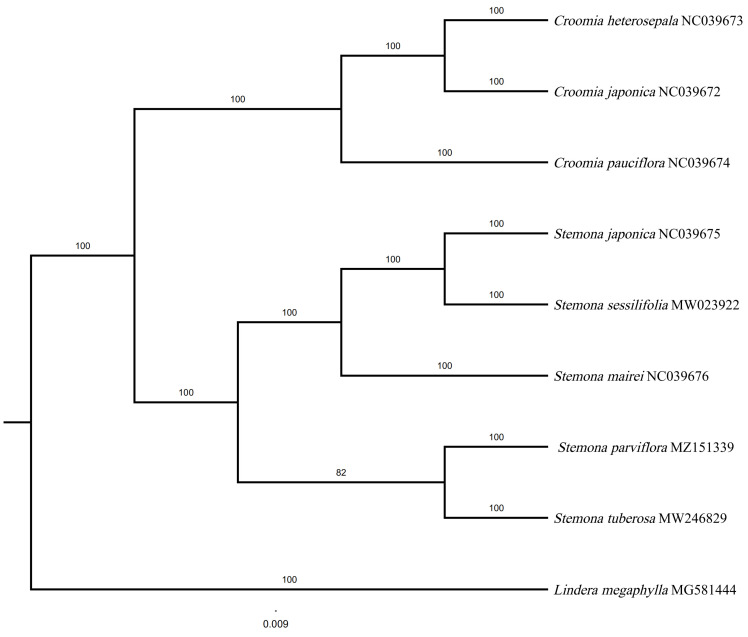
Molecular phylogenetic tree of *Stemona* using Bayesian inference method.

A BI tree was constructed based on 79 protein-coding genes using PhyloSuite (Figure 7). The tree received high bootstrap values in most nodes. In our phylogenetic tree, the monophyly of *Stemona* and *Croomia* received a high Bayesian posterior probability (100%). *Stemona* was sistered to *Croomia* with strong support (100%). The genus *Croomia* comprises three species worldwide (*C. heterosepala, C. japonica,* and *C. pauciflora*), of which only one species is distributed in China (*C. japonica*). *C. japonica* was closest to *C. heterosepala*. Five species in *Stemona* were mainly divided into two clades in the phylogenetic tree. *S. japonica*, *S. sessilifolia,* and *S. mairei* clustered to one clade. *S. parviflora* were sister to *S. tuberosa*, implying the close relationship between the two species.

### 3.8. Niche Analyses

**Figure 8:**
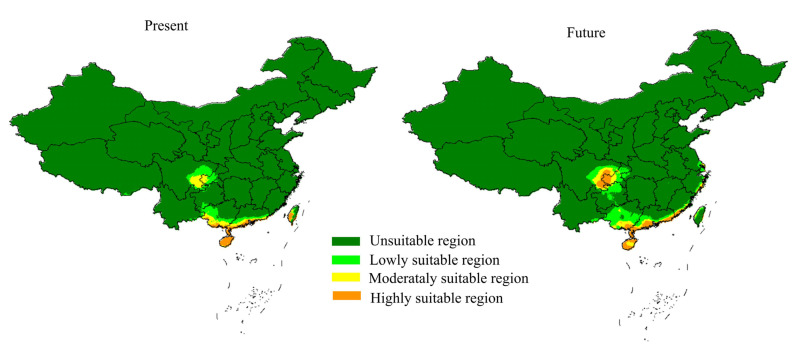
Potential distribution areas of *S. parviflora* in the current and future periods.

The AUC values of the MaxEnt results were all greater than 0.95, indicating that the model is very reliable. The results of MaxEnt show that the southwest of Guangdong and Guangxi, the southwest of Taiwan, and the vast majority of Hainan are highly suitable areas for *S. parviflora*, of which Hainan is the main suitable area. The future simulation results show that Hainan is still the main and highest fitness area of the *S. parviflora*, the southeast of Sichuan has changed from a medium fitness area to a high fitness area, the whole territory of Taiwan has changed to a low fitness area and below, and the southeast of Guangdong and its coastal areas are still high fitness areas of the *S. parviflora*. It is worth noting that the junction of Yunnan Province, Sichuan Province, and Chongqing is also highly suitable for the survival of *S. parviflora* (Figure 8).

## 4. Discussion

The structure of cp genomes was conservative in the genus *Stemona*, as well as in most land plants. The average size of cp genomes in *Stemona* was larger than its sister genus *Croomia* [30]. As most reported in other land plants, the LSC region has a relatively higher length variation compared with the IR and SSC regions, suggesting the IR and SSC regions were more conservative [31]. The GC content of the chloroplast genome ranged from 24.78% to 57.664%; the average of this occupied 36.82 % [32]. The GC content varied between different species in *Stemona*. For example, the GC content of *S. sessilifolia* and *S. japonica* was 38.0%; however, *S. parviflora*, *S. mairei*, and *S. tuberosa* had a GC content that accounted for 37.9%. The differentiation of GC content in the same genus was also reported in other studies [33]. Pseudogenization was commonly in the evolution of chloroplast genomes, such as the *accD*, *ccsA*, *ycf1*, *ycf2*, *ycf15*, *ycf86*, *rps19*, and *psbB* pseudogenes [32,34,35,36]. One pseudogene, ψ*ycf1*, was lost in the cp genome of *S. sessilifolia*, but was detected in four other species of *Stemona*. The pseudogenization of the *ycf1* gene may be interpreted as wastage in the course of species evolution, or as the fact that the species does not specifically need the gene. Moreover, the pseudogenization of *ycf1* was also detected in the genera *Paeonia* and *Aconitum* [37,38,39].

In the course of evolution, species will form a set of adaptive codon usage patterns. The codon usage bias is similar, indicating that species have similar living environmental or close genetic relationships [40,41]. Codon usage bias analyses have been widely used to investigate the evolution and phylogeny of land plants [42]. The value of relative synonymous codon usage (RSCU) was greater than one, indicating that there was codon usage bias. There were 29 to 30 codons greater than one among the five plastid genomes. A higher A/U content at the third codon position was detected with high RSCU values. These were different from other species that enriched AT at the third codon position [43]. The RSCU value of the genus *Stemona,* with almost no differences, was consistent with sect. *Moutan* species [37].

SSR has been used for population genetic and phylogenetic analyses between intraspecific and interspecific levels [44]. A total of 98, 106, 107, 96, and 111 SSRs were detected in *S. parviflora*, *S. sessilifloia*, *S. mairei*, *S. tuberose,* and the *S. japonica* chloroplast genome, respectively. Mononucleotides and dinucleotides were the most abundant repeat type in the plastid genome, as has been reported in other species [45]. In addition, we found that most of the SSR distributed in the LSC and SSC regions are consistent with the plastid genome of *Dracunculus* clade (Aroideae, Araceae) [46]. Long repeats sequences were also analyzed in this study. A total of 49 long repeats of four types were detected in the plastid genome of *S. parviflora*. As reported in other species, palindromic repeats were the most abundant repeat type [47].

The length of the plastid genome and genes in the IR/SSC and IR/LSC regions may change during the contraction and expansion of the IR region [48,49]. Prior research has demonstrated that the length of gene extent to IR/SSC and IR/LSC is associated with systematic analyses [50]. The content and length of the gene in the IR/LSC and IR/SSC boundary regions vary from congeneric species, as has been reported in the genus *Pedicularis* [36]. The genes *rps3* and *trnN* were more conservative compared with other genes in the genus *Stemona*. The genes *rpl22*, *ycf1*, and *psbA* from different species vary in gene length and distance from the IR/LSC and IR/SSC boundary regions. The genes *ndhF* and ψ*ycf1* had the same pattern in the rest of the four species, except for *S. sessilifloi*.

As reported in other angiosperm species, the LSC region was the most divergent, and IR regions were the most conservative [51]. The inter-gene spacer was more divergent compared with protein-coding sequences [52]. Twelve divergence regions were found in protein-coding sequences, including *rps16*, *trnQ-UUG*, *psbI*, *trnS-UGA*, *ycf3*, *trnS-GGA*, *trnL-UAA*, *psbJ*, *clpP*, *petD*, *rpl16*, *rpl22*, and *ycf1*. The *ycf1* gene had been used as DNA barcode for phylogenetic analyses [53]; moreover, significant variations were found in *ycf1* from many taxa, such as the *Quercus*, *Vicatia*, and *Curcuma* chloroplast genome [54,55]. In addition, we found the protein-coding gene *petD* was the most divergent compared among the five species of *Stemona*. *S. tuberosa* had little difference compared with *S. parvifloa* in the gene *petD*, which may be due to the close phylogenetic relationship between the two species.

The value of Ka/Ks was related to gene adaptive evolution, such as positive selection and purification selection effect [56]. The genes that were under positive selection might result from natural selection and adaptation to the living environment [57]. Seven genes (*atpI*, *ccsA*, *cemA*, *matK*, *ndhA*, *petA*, and *rpoC1*) were under positive selection in genus *Stemona*. The positive selection gene *matK* has been widely reported in other land plants, such as *Paphiopedilum* [58], *Chrysosplenium* [59], and *tribe Selineae* species [60]. The genes *atpI*, *ndhA*, and *petA* were related to photosynthesis; prior research has implied that light plays an important role in gene mutation rates, and these positive selection genes might be associated with sunlight conditions [59]. Furthermore, except for *rpoC1*, the rest of the six positive selection genes only appeared in one species, indicating that genes in different species of *Stemona* may have undergone different degrees of selective evolutionary pressure. There was no gene under positive selection between *S. parviflora* and *S. tuberosa*, which may result from the overlapping habitats of these two species. Thus, environmental selection pressure between the two species was not obvious.

In our phylogenetic tree, the monophyly of *Stenoma* and *Croomia* was well-supported. Our results also support *Stemona* as a sister to the genus *Croomia*, which is consistent with prior research [30,61]. Two main branches were shown in the phylogenetic tree and had a Bayesian posterior probability of 100%. Clade 1 contains *S. japonica*, *S. sessilifolia*, and *S. mairei*. *S. japonica* were closest to *S. sessilifolia*. In clade 2, *S. parviflora* and *S. tubersa* were clustered in a single branch, implying the close relationship between the two species. The *S. parviflora* and *S. tubeorsa* clustered in one branch, consisting of phylogenetic analyses that were based on *matK*, *rbcL*, and *psbK-psbI* cpDNA markets [3].

Species are fundamental to biodiversity [62]. Predicting the potential distribution range in currently and in the future will be significant for the protection and management of endangered species [63]. A population genetic study that investigated *S. parviflora* was last performed five years ago; besides, AFLP molecular markers are insufficient to represent the genetic diversity of species and are rarely used in current research [4]. Genome-wide SNP data should reassess the population diversity of *S. parviflora*. Some protective measures have been proposed by previous research, such as seed germination, tissue culture, introduction and conservation, and pollination by the insect protection [4]. The highly suitable region of *S. parviflora* was not provided in the previous study, which makes it difficult for current introduction and cultivation measures. Fragmentation of the distribution range leads to *S. parviflora* being more vulnerable to threats. In addition, the actual distribution area is narrower than the simulation results. Limited by seed dispersal mechanisms (mainly by ants) [2], the spread distance of *S. parviflora* was is narrow. Although the niche simulation results show that most areas in Southwest Guangdong and Hainan are highly suitable for the survival of *S. parviflora*, due to the limited dispersal capacity of *S. parviflora*, it is necessary to carry out artificial grafts to expand the survival areas of *S. parviflora*. Furthermore, in the medium- and above-suitable regions revealed in this study, the whole territory of Taiwan has changed to a low fitness area and below in the 2050 s, which may not be suitable for the introduction and cultivation of *S. parviflora*. The suitability of the junction between Yunnan Province, Sichuan Province, and Chongqing for the survival of *S. parviflora* needs more transplant and cultivation measures to be verified. Overall, our results provide multiple highly suitable areas for *S. parviflora*, which may play a crucial role in current and future conservation measures.

## 5. Conclusions

In this study, the chloroplast genome of the endangered species *S. parviflora* was assembled and compared with four other *Stemona* species. The structure of the chloroplast genome was extremely conservative in the genus *Stemona*. Long repeat and SSR sequences were detected in this study, which may be used for population genetic analyses. We also compared the IR/LSC and IR/SSC boundary region, code usage bias, and the whole plastid genome. Seven genes (*atpI*, *ccsA*, *cemA*, *matK*, *ndhA*, *petA*, and *rpoC1*) were detected under positive selection. Phylogenetic analyses implied that *S. parviflora* was closest to *S. tubersa*. Niche simulation results showed that the southeast of Guangdong, the majority of Hainan, and Southwest Taiwan were highly suitable for *S. parviflora* survival in the current period. The changes in highly suitable areas in the future (2050s) are also discussed in this study.

## Figures and Tables

**Table 1:** Comparative analysis of the complete chloroplast genome of genus *Stemona*.

Species	Length	LSC	SSC	IR	GC	Number of Gene	tRNA	rRNA	Pesedogene
*S. japonica*	154,224 bp	82,117 bp	17,943 bp	27,082 bp	38.0%	134	38	8	1
*S. sessilifolia*	154,037 bp	81,949 bp	17,952 bp	27,068 bp	38.0%	133	38	8	0
*S. parviflora*	154,552 bp	82,410 bp	17,966 bp	27,088 bp	37.9%	134	38	8	1
*S. tuberosa*	154,374 bp	82,305 bp	17,929 bp	27,070 bp	37.9%	134	38	8	1
*S. mairei*	154,307 bp	82,254 bp	17,889 bp	27,082 bp	38.0%	134	38	8	1

## Data Availability

The chloroplast genome sequences have been deposited in GenBank under the accession number: MZ151339. Row data are available at SRA under the accession number: PRJNA850147.

## Supplementary Materials

The following supporting information can be downloaded at: https://www.mdpi.com/article/10.3390/genes13081361/s1, Table S1 Effective latitude and longitude coordinates of *S. parviflora* used in this study, Table S2 Correlation of 19 climatic factors, Table S3 Relative synonymous codon usage (RSCU) of 79 protein-coding genes of *Stemona*.
