# Supplementary material for: The Complete Chloroplast Genome of Endangered Species Stemona parviflora: Insight into the Phylogenetic Relationship and Conservation Implications
Source: Genes (Basel). 2022 Jul 29;13(8):1361. doi: 10.3390/genes13081361 (PMC9407434; doi:10.3390/genes13081361)
Supplement: Supplementary file 1 [file genes-13-01361-s001.zip › Table S1.pdf]

Table S1 Effective latitude and longitude coordinates of *S. parviflora* used in this study

Latitude (N)	Longitude (E)
19°55´40.43"	110°20´24.14"
29°06´30.9"	107°43´22.4"
19°01´46"	109°23´39"
19°53'34.8"	110°17'32.28"
18°40′06.52"	109°55′20.38"
18°30′11.53"	109°13′39.22"
19°00′29.28"	109°21′56.42"
18°30′16.57"	110°01′17.77"
19°01′46.02"	109°23′39.05"
18°52'54.88"	109°40'1.88"
19°13'46.92"	110°28'19.49"
18°48'8.29"	110°25'26.73"
20º16′30.82"	110º16′9.98"
18º24′39.29"	109º40′19.79"
19º47′02.41"	109º46′28.58"
19° 6'24.15"	108°49'33.66"
19º13′1.44"	109º47′5.41"
18°42'25.15"	108°49'46.85"
19º55′42.54”	110º12′56.32"
