# Supplementary material for: The Complete Chloroplast Genome of Endangered Species Stemona parviflora: Insight into the Phylogenetic Relationship and Conservation Implications
Source: Genes (Basel). 2022 Jul 29;13(8):1361. doi: 10.3390/genes13081361 (PMC9407434; doi:10.3390/genes13081361)
Supplement: Supplementary file 1 [file genes-13-01361-s001.zip › Table S2.pdf]

Table S2 Correlation of 19 climatic factors

	bio1	bio2	bio3	bio4	bio5	bio6	bio7	bio8	bio9	bio10	bio11	bio12	bio13	bio14	bio15	bio16	bio17	bio18	bio19
bio1		0.578	0.700	-0.545	0.967	0.974	-0.369	0.945	0.918	0.976	0.980	0.540	0.541	0.316	-0.389	0.562	0.332	0.518	0.317
bio2[a,b]			0.431	-0.151	0.649	0.479	0.126	0.549	0.516	0.603	0.527	0.014	0.098	-0.109	0.056	0.098	-0.113	0.094	-0.091
bio3[b]				-0.852	0.551	0.799	-0.735	0.527	0.782	0.563	0.798	0.637	0.632	0.352	-0.051	0.641	0.380	0.478	0.476
bio4[b]					-0.322	-0.713	0.957	-0.303	-0.732	-0.352	-0.699	-0.557	-0.561	-0.288	0.026	-0.563	-0.319	-0.359	-0.430
bio5						0.887	-0.124	0.970	0.826	0.997	0.901	0.431	0.437	0.253	-0.409	0.459	0.262	0.457	0.223
bio6[a,b]							-0.569	0.865	0.955	0.904	0.998	0.608	0.601	0.359	-0.351	0.620	0.381	0.529	0.397
bio7[a]								-0.130	-0.581	-0.167	-0.539	-0.538	-0.513	-0.320	0.026	-0.515	-0.351	-0.321	-0.455
bio8[a]									0.755	0.976	0.875	0.472	0.469	0.292	-0.466	0.493	0.303	0.529	0.232
bio9										0.838	0.956	0.527	0.528	0.290	-0.288	0.542	0.313	0.405	0.374
bio10											0.915	0.460	0.460	0.279	-0.429	0.483	0.289	0.483	0.246
bio11												0.590	0.593	0.335	-0.334	0.611	0.357	0.523	0.372
bio12[a]													0.913	0.749	-0.326	0.937	0.782	0.836	0.763
bio13[b]														0.479	-0.106	0.993	0.513	0.784	0.622
bio14[a,b]															-0.471	0.515	0.993	0.625	0.678
bio15[a]																-0.158	-0.484	-0.302	-0.298
bio16																	0.549	0.806	0.645
bio17																		0.644	0.707
bio18[a,b]																			0.423
bio19[a,b]																			

Note: [a]； selected climatic factors for current distribution area simulation; [b]: selected climatic factors for future distribution area simulation
