# Supplementary material for: The Complete Chloroplast Genome of Endangered Species Stemona parviflora: Insight into the Phylogenetic Relationship and Conservation Implications
Source: Genes (Basel). 2022 Jul 29;13(8):1361. doi: 10.3390/genes13081361 (PMC9407434; doi:10.3390/genes13081361)
Supplement: Supplementary file 1 [file genes-13-01361-s001.zip › Table S3.pdf]

Table S3 Relative synonymous codon usage (RSCU) of 79 protein-coding genes of *Stemona*

S. japonica		*S. tuberosa*		*S. parviflora*		*S. mairei*		*S. sessilifolia*	
Code (Amino acid)	Number (RSCU)	Code (Amino acid)	Number (RSCU)	Code (Amino acid)	Number (RSCU)	Code (Amino acid)	Number (RSCU)	Code (Amino acid)	Number (RSCU)
UUU(F)	797.0(1.25)	UUU(F)	799.0(1.26)	UUU(F)	799.0(1.25)	UUU(F)	800.0(1.25)	UUU(F)	797.0(1.25)
UUC(F)	476.0(0.75)	UUC(F)	470.0(0.74)	UUC(F)	479.0(0.75)	UUC(F)	476.0(0.75)	UUC(F)	477.0(0.75)
UUA(L)	746.0(1.90)	UUA(L)	754.0(1.92)	UUA(L)	740.0(1.89)	UUA(L)	747.0(1.90)	UUA(L)	744.0(1.90)
UUG(L)	497.0(1.27)	UUG(L)	495.0(1.26)	UUG(L)	501.0(1.28)	UUG(L)	496.0(1.26)	UUG(L)	494.0(1.26)
CUU(L)	499.0(1.27)	CUU(L)	498.0(1.27)	CUU(L)	500.0(1.27)	CUU(L)	501.0(1.28)	CUU(L)	498.0(1.27)
CUC(L)	151.0(0.38)	CUC(L)	148.0(0.38)	CUC(L)	147.0(0.37)	CUC(L)	147.0(0.37)	CUC(L)	150.0(0.38)
CUA(L)	321.0(0.82)	CUA(L)	319.0(0.81)	CUA(L)	322.0(0.82)	CUA(L)	318.0(0.81)	CUA(L)	319.0(0.81)
CUG(L)	143.0(0.36)	CUG(L)	145.0(0.37)	CUG(L)	145.0(0.37)	CUG(L)	145.0(0.37)	CUG(L)	144.0(0.37)
AUU(I)	937.0(1.44)	AUU(I)	947.0(1.44)	AUU(I)	949.0(1.44)	AUU(I)	936.0(1.44)	AUU(I)	939.0(1.44)
AUC(I)	381.0(0.58)	AUC(I)	386.0(0.59)	AUC(I)	384.0(0.58)	AUC(I)	388.0(0.60)	AUC(I)	383.0(0.59)
AUA(I)	636.0(0.98)	AUA(I)	639.0(0.97)	AUA(I)	638.0(0.97)	AUA(I)	629.0(0.97)	AUA(I)	634.0(0.97)
AUG(M)	544.0(1.00)	AUG(M)	536.0(1.00)	AUG(M)	535.0(1.00)	AUG(M)	540.0(1.00)	AUG(M)	543.0(1.00)
GUU(V)	471.0(1.46)	GUU(V)	476.0(1.48)	GUU(V)	475.0(1.47)	GUU(V)	472.0(1.46)	GUU(V)	471.0(1.46)
GUC(V)	162.0(0.50)	GUC(V)	157.0(0.49)	GUC(V)	158.0(0.49)	GUC(V)	162.0(0.50)	GUC(V)	162.0(0.50)
GUA(V)	471.0(1.46)	GUA(V)	473.0(1.47)	GUA(V)	472.0(1.46)	GUA(V)	474.0(1.47)	GUA(V)	470.0(1.46)
GUG(V)	188.0(0.58)	GUG(V)	183.0(0.57)	GUG(V)	185.0(0.57)	GUG(V)	185.0(0.57)	GUG(V)	189.0(0.59)
UCU(S)	497.0(1.72)	UCU(S)	494.0(1.71)	UCU(S)	498.0(1.73)	UCU(S)	498.0(1.72)	UCU(S)	499.0(1.72)
UCC(S)	273.0(0.94)	UCC(S)	272.0(0.94)	UCC(S)	268.0(0.93)	UCC(S)	272.0(0.94)	UCC(S)	272.0(0.94)
UCA(S)	352.0(1.22)	UCA(S)	351.0(1.22)	UCA(S)	353.0(1.22)	UCA(S)	349.0(1.20)	UCA(S)	350.0(1.21)

UCG(S)	162.0(0.56)	UCG(S)	159.0(0.55)	UCG(S)	155.0(0.54)	UCG(S)	161.0(0.56)	UCG(S)	161.0(0.56)
CCU(P)	368.0(1.54)	CCU(P)	368.0(1.55)	CCU(P)	365.0(1.54)	CCU(P)	370.0(1.55)	CCU(P)	367.0(1.54)
CCC(P)	210.0(0.88)	CCC(P)	203.0(0.85)	CCC(P)	207.0(0.87)	CCC(P)	205.0(0.86)	CCC(P)	209.0(0.87)
CCA(P)	250.0(1.04)	CCA(P)	250.0(1.05)	CCA(P)	251.0(1.06)	CCA(P)	252.0(1.05)	CCA(P)	251.0(1.05)
CCG(P)	129.0(0.54)	CCG(P)	130.0(0.55)	CCG(P)	127.0(0.53)	CCG(P)	129.0(0.54)	CCG(P)	129.0(0.54)
ACU(T)	459.0(1.55)	ACU(T)	456.0(1.55)	ACU(T)	456.0(1.55)	ACU(T)	459.0(1.55)	ACU(T)	457.0(1.54)
ACC(T)	218.0(0.74)	ACC(T)	212.0(0.72)	ACC(T)	212.0(0.72)	ACC(T)	213.0(0.72)	ACC(T)	217.0(0.73)
ACA(T)	384.0(1.30)	ACA(T)	390.0(1.32)	ACA(T)	386.0(1.31)	ACA(T)	386.0(1.31)	ACA(T)	386.0(1.30)
ACG(T)	125.0(0.42)	ACG(T)	121.0(0.41)	ACG(T)	125.0(0.42)	ACG(T)	123.0(0.42)	ACG(T)	124.0(0.42)
GCU(A)	569.0(1.81)	GCU(A)	568.0(1.80)	GCU(A)	566.0(1.80)	GCU(A)	568.0(1.80)	GCU(A)	570.0(1.81)
GCC(A)	189.0(0.60)	GCC(A)	192.0(0.61)	GCC(A)	191.0(0.61)	GCC(A)	193.0(0.61)	GCC(A)	189.0(0.60)
GCA(A)	351.0(1.12)	GCA(A)	350.0(1.11)	GCA(A)	350.0(1.11)	GCA(A)	352.0(1.12)	GCA(A)	352.0(1.12)
GCG(A)	148.0(0.47)	GCG(A)	149.0(0.47)	GCG(A)	150.0(0.48)	GCG(A)	148.0(0.47)	GCG(A)	147.0(0.47)
UAU(Y)	681.0(1.60)	UAU(Y)	683.0(1.60)	UAU(Y)	682.0(1.60)	UAU(Y)	682.0(1.61)	UAU(Y)	681.0(1.60)
UAC(Y)	168.0(0.40)	UAC(Y)	170.0(0.40)	UAC(Y)	170.0(0.40)	UAC(Y)	167.0(0.39)	UAC(Y)	170.0(0.40)
UAA(*)	33.0(1.25)	UAA(*)	33.0(1.25)	UAA(*)	33.0(1.25)	UAA(*)	33.0(1.25)	UAA(*)	33.0(1.25)
UAG(*)	23.0(0.87)	UAG(*)	23.0(0.87)	UAG(*)	23.0(0.87)	UAG(*)	23.0(0.87)	UAG(*)	23.0(0.87)
CAU(H)	431.0(1.57)	CAU(H)	437.0(1.58)	CAU(H)	434.0(1.57)	CAU(H)	432.0(1.57)	CAU(H)	428.0(1.57)
CAC(H)	117.0(0.43)	CAC(H)	115.0(0.42)	CAC(H)	118.0(0.43)	CAC(H)	117.0(0.43)	CAC(H)	117.0(0.43)
CAA(Q)	609.0(1.53)	CAA(Q)	609.0(1.53)	CAA(Q)	605.0(1.53)	CAA(Q)	607.0(1.53)	CAA(Q)	607.0(1.53)
CAG(Q)	186.0(0.47)	CAG(Q)	185.0(0.47)	CAG(Q)	186.0(0.47)	CAG(Q)	189.0(0.47)	CAG(Q)	186.0(0.47)
AAU(N)	804.0(1.54)	AAU(N)	801.0(1.54)	AAU(N)	804.0(1.55)	AAU(N)	805.0(1.55)	AAU(N)	804.0(1.55)

AAC(N)	239.0(0.46)	AAC(N)	237.0(0.46)	AAC(N)	233.0(0.45)	AAC(N)	235.0(0.45)	AAC(N)	235.0(0.45)
AAA(K)	851.0(1.47)	AAA(K)	853.0(1.47)	AAA(K)	853.0(1.47)	AAA(K)	856.0(1.48)	AAA(K)	848.0(1.47)
AAG(K)	303.0(0.53)	AAG(K)	306.0(0.53)	AAG(K)	306.0(0.53)	AAG(K)	301.0(0.52)	AAG(K)	302.0(0.53)
GAU(D)	743.0(1.59)	GAU(D)	736.0(1.58)	GAU(D)	740.0(1.58)	GAU(D)	735.0(1.59)	GAU(D)	742.0(1.59)
GAC(D)	193.0(0.41)	GAC(D)	195.0(0.42)	GAC(D)	198.0(0.42)	GAC(D)	192.0(0.41)	GAC(D)	193.0(0.41)
GAA(E)	903.0(1.50)	GAA(E)	902.0(1.49)	GAA(E)	903.0(1.50)	GAA(E)	900.0(1.49)	GAA(E)	903.0(1.50)
GAG(E)	304.0(0.50)	GAG(E)	307.0(0.51)	GAG(E)	302.0(0.50)	GAG(E)	310.0(0.51)	GAG(E)	304.0(0.50)
UGU(C)	199.0(1.49)	UGU(C)	199.0(1.49)	UGU(C)	198.0(1.48)	UGU(C)	199.0(1.49)	UGU(C)	198.0(1.48)
UGC(C)	69.0(0.51)	UGC(C)	68.0(0.51)	UGC(C)	69.0(0.52)	UGC(C)	68.0(0.51)	UGC(C)	70.0(0.52)
UGA(*)	23.0(0.87)	UGA(*)	23.0(0.87)	UGA(*)	23.0(0.87)	UGA(*)	23.0(0.87)	UGA(*)	23.0(0.87)
UGG(W)	402.0(1.00)	UGG(W)	401.0(1.00)	UGG(W)	402.0(1.00)	UGG(W)	401.0(1.00)	UGG(W)	402.0(1.00)
CGU(R)	321.0(1.39)	CGU(R)	316.0(1.37)	CGU(R)	314.0(1.36)	CGU(R)	319.0(1.37)	CGU(R)	320.0(1.38)
CGC(R)	93.0(0.40)	CGC(R)	92.0(0.40)	CGC(R)	94.0(0.41)	CGC(R)	91.0(0.39)	CGC(R)	94.0(0.40)
CGA(R)	294.0(1.27)	CGA(R)	295.0(1.28)	CGA(R)	295.0(1.27)	CGA(R)	296.0(1.27)	CGA(R)	293.0(1.26)
CGG(R)	107.0(0.46)	CGG(R)	106.0(0.46)	CGG(R)	109.0(0.47)	CGG(R)	110.0(0.47)	CGG(R)	108.0(0.46)
AGU(S)	356.0(1.23)	AGU(S)	356.0(1.23)	AGU(S)	356.0(1.23)	AGU(S)	359.0(1.24)	AGU(S)	357.0(1.23)
AGC(S)	98.0(0.34)	AGC(S)	101.0(0.35)	AGC(S)	102.0(0.35)	AGC(S)	99.0(0.34)	AGC(S)	98.0(0.34)
AGA(R)	432.0(1.86)	AGA(R)	435.0(1.88)	AGA(R)	433.0(1.87)	AGA(R)	431.0(1.86)	AGA(R)	434.0(1.87)
AGG(R)	143.0(0.62)	AGG(R)	144.0(0.62)	AGG(R)	144.0(0.62)	AGG(R)	146.0(0.63)	AGG(R)	145.0(0.62)
GGU(G)	525.0(1.33)	GGU(G)	528.0(1.34)	GGU(G)	526.0(1.34)	GGU(G)	529.0(1.34)	GGU(G)	525.0(1.34)
GGC(G)	147.0(0.37)	GGC(G)	148.0(0.38)	GGC(G)	150.0(0.38)	GGC(G)	147.0(0.37)	GGC(G)	147.0(0.37)
GGA(G)	623.0(1.58)	GGA(G)	618.0(1.57)	GGA(G)	614.0(1.56)	GGA(G)	621.0(1.57)	GGA(G)	620.0(1.58)

GGG(G)	284.0(0.72)	GGG(G)	279.0(0.71)	GGG(G)	285.0(0.72)	GGG(G)	281.0(0.71)	GGG(G)	281.0(0.71)
